# Identification and Characterization of Genes Involved in Benzylisoquinoline Alkaloid Biosynthesis in *Coptis* Species

**DOI:** 10.3389/fpls.2018.00731

**Published:** 2018-06-04

**Authors:** Si-Mei He, Yan-Li Liang, Kun Cong, Geng Chen, Xiu Zhao, Qi-Ming Zhao, Jia-Jin Zhang, Xiao Wang, Yang Dong, Jian-Li Yang, Guang-Hui Zhang, Zhi-Long Qian, Wei Fan, Sheng-Chao Yang

**Affiliations:** ^1^State Key Laboratory of Conservation and Utilization of Bio-Resources in Yunnan, The Key Laboratory of Medicinal Plant Biology of Yunnan Province, National and Local Joint Engineering Research Center on Germplasm Innovation and Utilization of Chinese Medicinal Materials in Southwest China, Yunnan Agricultural University, Kunming, China; ^2^State Key Laboratory of Genetic Resources and Evolution, Kunming Institute of Zoology, Chinese Academy of Sciences, Kunming, China; ^3^Kunming College of Life Science, University of Chinese Academy of Sciences, Kunming, China; ^4^Province Key Laboratory, Biological Big Data College, Yunnan Agricultural University, Kunming, China; ^5^State Key Laboratory of Plant Physiology and Biochemistry, College of Life Sciences, Zhejiang University, Hangzhou, China

**Keywords:** benzylisoquinoline alkaloid, biosynthesis, *Coptis*, *O*-methyltransferases, transcriptome

## Abstract

The dried rhizomes of *Coptis chinensis* have been extensively used in heat clearing, dampness drying, fire draining, and detoxification by virtue of their major bioactive components, benzylisoquinoline alkaloids (BIAs). However, *C. teeta* and *C. chinensis* are occasionally interchanged, and current understanding of the molecular basis of BIA biosynthesis in these two species is limited. Here, berberine, coptisine, jatrorrhizine, and palmatine were detected in two species, and showed the highest contents in the roots, while epiberberine were found only in *C. chinensis*. Comprehensive transcriptome analysis of the roots and leaves of *C. teeta* and *C*. *chinensis*, respectively, identified 53 and 52 unigenes encoding enzymes potentially involved in BIA biosynthesis. By integrating probable biosynthetic pathways for BIAs, the jatrorrhizine biosynthesis ill-informed previously was further characterized. Two genes encoding norcoclaurine/norlaudanosoline 6-*O*-methyltransferases (*Cc6OMT1* and *Cc6OMT2*) and one gene encoding norcoclaurine-7OMT (*Ct7OMT*) catalyzed enzymatically *O-*methylate (*S*)-norcoclaurine at C6 that yield (*S*)-coclaurine, along with a smaller amount of *O*-methylation occurred at C7, thereby forming its isomer (isococlaurine). In addition, scoulerine 9-OMT (CtSOMT) was determined to show strict substrate specificity, targeting (*S*)-scoulerine to yield (*S*)-tetrahydrocolumbamine. Taken together, the integration of the transcriptome and enzyme activity assays further provides new insight into molecular mechanisms underlying BIA biosynthesis in plants and identifies candidate genes for the study of synthetic biology in microorganisms.

## Introduction

*Coptis chinensis* is a perennial herb that belongs to the family Ranunculaceae. Among 16 species distributed all over the world, 6 distribute in Sichuan, Guizhou, Yunnan, and Shanxi provinces of western China (Zhang Y. et al., 2015; Xue et al., 2017). For centuries, its dried rhizomes have been widely used in traditional Chinese medicine (TCM) (Kamath et al., 2009; Xiang et al., 2016) for heat clearing, dampness drying, fire draining, and detoxification (Zhang et al., 2011; Yang et al., 2017). Currently, *C. chinensis* is a commonly used TCM with definitive curative effects in clinic, and 108 patented drugs have been produced from *C. chinensis* in China (Feng et al., 2008; Wang et al., 2014). Due to overexploitation, the wild resources of this herb are now endangered. To meet the ever increasing demand of this herb, studies on the biosynthesis of the bioactive components of *C. chinensis* are important to ensure the reasonable utilization of resources and the use of synthetic biology through microorganism engineering.

The major active components in *C. chinensis* are benzylisoquinoline alkaloids (BIAs) (Kamath et al., 2009; Chen et al., 2017). Of great interest in this field are the identification of effective separation methods and assessment of their efficacy in the treatment of various diseases (Tjong et al., 2011; Zhang et al., 2011; Cui et al., 2016; Friedemann et al., 2016; Kim et al., 2016). Chemical investigations have determined that the major BIAs in *C. chinensis* are protoberberine alkaloids, which include berberine, palmatine, jatrorrhizine, coptisine, columbamine, magnoflorine, and epiberberine (Lv et al., 2016; Yang et al., 2017), with berberine as the predominant component (Tang et al., 2009; Ye et al., 2009; Lv et al., 2016). These compounds have been shown to be efficacious in antiviral, anti-inflammatory, and antimicrobial activity, for dispelling dampness, for removing toxicosis, and in detoxification (Tjong et al., 2011; Zhang et al., 2011; Cui et al., 2016; Friedemann et al., 2016; Kim et al., 2016). However, current understanding of the biosynthesisof BIAs in *C. chinensis* remains limited due to insufficient genomic resources.

**FIGURE 1:**
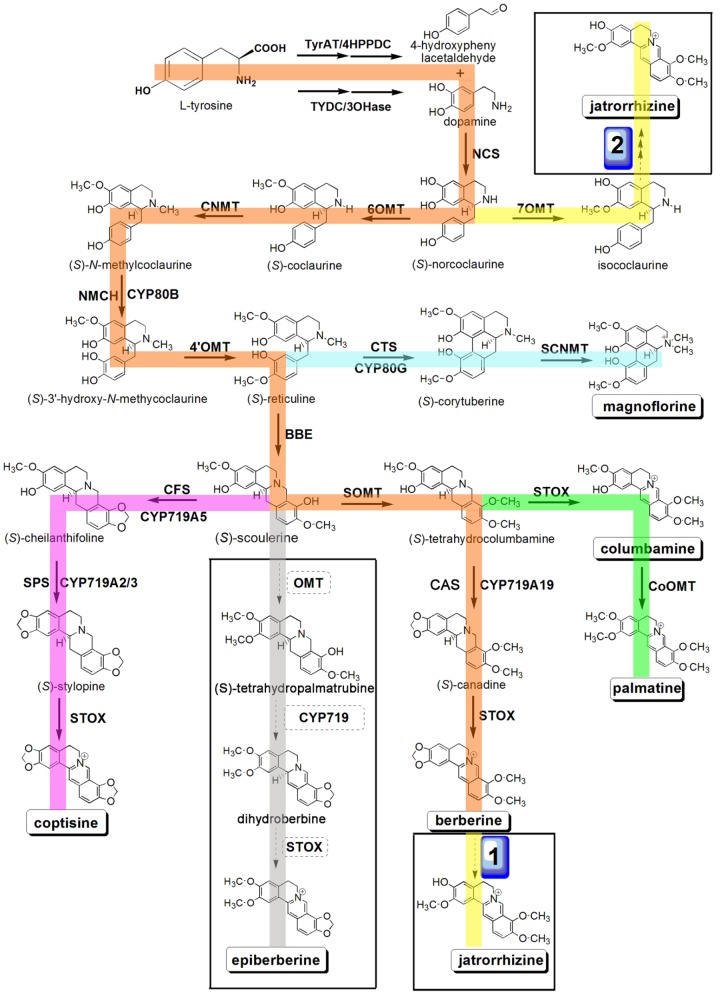
Putative pathways for BIA biosynthesis in *C. teeta* and *C*. *chinensis*. Enzymes found in this study are boxed. Abbreviations: TyrAT, L-tyrosine aminotransferase; 4HPPDC, 4-hydroxyphenylpuruvate decarboxylase; TYDC, tyrosine decarboxylase; 3OHase, tyrosine/tyramine 3-hydroxylase; NCS, (*S*)-norcoclaurine synthase; 6OMT, norcoclaurine 6-*O*-methyltransferase; CNMT, (*S*)-coclaurine *N*-methyltransferase; NMCH, *N*-methylcoclaurine 3^′^-hydroxylase; 4^′^OMT, 3^′^-hydroxy-*N*-methylcoclaurine 4^′^-*O*-methyltransferase; BBE, berberine bridge enzyme; CTS, corytuberine synthase; SCNMT, (*S*)-corytuberine-*N*-methyltransferase; SOMT, (*S*)-scoulerine 9-*O*-methyltransferase; CAS, (*S*)-canadine synthase; STOX, (*S*)-tetrahydroprotoberberine oxidase; CoOMT, columbamine *O*-methyltransferase; CFS, (*S*)-cheilanthifoline synthase; SPS, (*S*)-stylopine synthase.

The biosynthetic pathways for several BIAs have been reported in a number of plant species, such as berberine in *C. japonica*, sanguinarine in *Eschscholzia californica*, and morphine in *Papaver somniferum*, and many enzymes involved in BIA biosynthesis in these plants have been characterized (Morishige et al., 2010; Hagel and Facchini, 2013; Sato, 2013). The biosynthesis of BIAs begins with the conversion of L-tyrosine to dopamine and 4-hydroxyphenylacetaldehyde, which are then condensed to (*S*)-norcoclaurine by (*S*)-norcoclaurine synthase (NCS) (Samanani and Facchini, 2002; Samanani et al., 2004; Minami et al., 2007). Three methyltransferases [(*S*)-norcoclaurine/norlaudanosoline 6-*O*-methyltransferase (6OMT), (*S*)-coclaurine-*N*-methyltransferase (CNMT), and (*S*)-3^′^-hydroxy-*N*-methylcoclaurine-4^′^-*O*-methyltransferase (4^′^OMT)] and one cytochrome P450 [P450, (*S*)-*N*-methylcoclaurine 3^′^-hydroxylase (NMCH)] are involved in catalyzing the conversion of (*S*)-norcoclaurine to (*S*)-reticuline, which is a central intermediate for the production of different BIAs, including protoberberine, benzophenanthridine, and morphinan alkaloids (Morishige et al., 2000; Choi et al., 2002; Samanani et al., 2006; Sato et al., 2007; Ziegler and Facchini, 2008; Hagel and Facchini, 2013; Beaudoin and Facchini, 2014; Yamada et al., 2015). BIA biosynthesis consists of several steps that involve oxidation reactions catalyzed by P450s and *O*-methylation processes catalyzed by members of the OMT family that participate in the synthesis of the intermediate product, (*S*)-reticuline, followed by multistep transformations that yield different end-products (Chapple, 1998; Mizutani and Sato, 2011; Dang and Facchini, 2012; Takemura et al., 2013; Chang et al., 2015) (**Figure 1**).

The identification and characterization of related genes involved in the synthesis of bioactive components is essential for understanding the molecular mechanism of biosynthetic pathways. With the rapid development of RNA sequencing technology, it is easy to capture the candidate genes at a genome-wide scale. In a recent study, Chen et al. (2017) analyzed major components of *C*. *chinensis* from three biotopes; namely, Zhenping, Zunyi, and Shizhu, and found that berberine was highest in concentration, followed sequentially by coptisine, palmatine, and epiberberine in all three accessions. Among these accessions, the drug quality of the accession from Shizhu may be the highest (Fan et al., 2012). Further transcriptomic investigation and correlation analysis of chemical components revealed four candidate genes encoding aspartate aminotransferase, polyphenol oxidase, primary-amine oxidase, and tyrosine decarboxylase (TYDC). However, functional characterization of these candidate genes has not been performed to date.

Of the six species of *C. chinensis* distributed in China, *C. chinensis* Franch., *C. deltoid* C.Y. Chen et Hsiao, and *C. teeta* Wall. are respectively called “Weilian,” “Yalian,” and “Yunlian” in the Chinese Pharmacopeia (version 2010) (Yang et al., 2012; Li et al., 2013). Among these, *C. chinensis* is the most important raw material because it is widely distributed and has a high yield, whereas *C. teeta* is an endangered species (Zhang Y. et al., 2015). Previously, people thought that the three species could be used interchangeably because of their similar phytochemical contents (mainly alkaloids) (Lv et al., 2016). However, accumulating evidence shows that they differ in their pharmacological activities. For example, Feng et al. (2011) determined that the rhizomes of *C. chinensis* have higher antibacterial activity than *C. deltoidea* and *C. teeta.* Therefore, analysis of their chemical compositions is essential for promoting their appropriate pharmacological use.

In the present study, we carried out a comprehensive transcriptome study of *C. chinensis* and *C. teeta* by means of next-generation RNA-seq technology. Combining our present results with previous reports (Rüffer et al., 1983; Ikezawa et al., 2008; Ziegler et al., 2009; Hagel et al., 2015; He et al., 2017), we propose the biosynthetic pathways for berberine, palmatine, jatrorrhizine, coptisine, columbamine, magnoflorine, and epiberberine, and identify candidate genes that are involved in the biosynthesis of BIAs. Moreover, we characterized 6OMT and norcoclaurine-7OMT (7OMT), which catalyze *O*-methylation at C6 on (*S*)-norcoclaurine to yield (*S*)-coclaurine, along with a smaller amount of *O*-methylation at C7, thereby forming its isomer, isococlaurine. SOMT 9-*O*-methylates (*S*)-scoulerine specifically to yield (*S*)-tetrahydrocolumbamine. Taken together, our study further promotes the molecular and biochemical understanding of BIA production in *Coptis* and provides substantial genetic resources for further research.

## Materials and Methods

### Plant Materials

*C. teeta* was collected from Nujiang, Yunnan Province (26°54^′^N, 98°97^′^E and altitude: 2771 m), while *C*. *chinensis* was collected from an area managed by the Guangxi Botanical Garden of Medicinal Plants in Nanning, China. The roots and leaves were harvested separately, immediately frozen in liquid nitrogen, and stored at -80^°^C until use.

### cDNA Library Construction, Sequencing and *de Novo* Assembly

Total RNA of roots and leaves from two *Coptis* species were extracted using RNeasy Plant Mini Kit (Qiagen, Hilden, Germany). The quantity and quality of total RNA were detected by the NanoDrop system (Thermo Scientific, United States) and gel electrophoresis, respectively. Then, at least 4 μg of total RNA samples were used to construct mRNA libraries and deep sequencing by NEBNext^®^ Ultra^TM^ RNA Library Prep Kit and Illumina sequencing on a Hiseq 2000 platform, respectively. After RNA-seq, raw reads were firstly transformed into clean reads by removing adaptors, low-quality and unknown nucleotides. Then, the clean reads were *de novo* assembled using the Trinity program (k-mer = 25, group pairs distance = 300) with default parameters **(**Liscombe et al., 2005).

### Functional Annotation, CDS Prediction and Phylogenetic Analysis

For functional annotations, the assembled unigenes were blasted against public databases (*E*-value < 1 × 10^-5^), including the NCBI non-redundant protein (NR) database^1^, the SwissProt database^2^, the euKaryotic Ortholog Groups (KOG) database^3^, the Kyoto Encyclopedia of Genes and Genomes (KEGG) database^4^, and the protein family (Pfam) database (version 26.0) (Finn et al., 2014). Based on NR database annotation, GO unigene annotations were obtained by Blast2GO program (Conesa et al., 2005). Then, GO functional classification was performed by WEGO software (Ye et al., 2006). Finally, CDSs of unigenes were predicted using BLSATX and ESTscan (Iseli et al., 1999). In addition, a neighbor-joining tree was built by Clustal X 2.0 and MEGA 5.0 with deduced amino acid of OMTs from two *Coptis* and other plants.

### High-Performance Liquid Chromatography Analysis

0.2 g dried powder of two *Coptis* leaves, roots and rhizomes was respectively extracted with 50 mL of hydrochloric acid-methanol mixed liquor (1:100, v/v) for 30 min, and sonicated for 30 min. For determining the main bioactive components of two *Coptis* species, an Agilent 1260 High-performance liquid chromatography (HPLC) system (Agilent Technologies, Santa Clara, CA, United States) was used. Chromatographic separation was performed on the chromatographic column Agilent Zorbax SB-C 18 (250 mm × 4.6 mm, 5 μm, Agilent Technologies) at a column temperature of 30^°^C, the flow rate was fixed at 1 mL/min. The mobile phase consisted of acetonitrile-0.05mol/L potassium dihydrogen phosphate solution (50:50, v/v) containing 0.1% sodium dodecyl sulfate, and separation was achieved by an isocratic elution. Detection was performed at 345 nm (Zhang G.H. et al., 2015; He et al., 2017). The content of berberine, coptisine, jatrorrhizine, palmatine, and epiberberine were calculated from standard curves. Authentic berberine, palmatine, jatrorrhizine, coptisine and epiberberine were purchased from JK chemical (Beijing, China).

### Recombinant Protein Expression and Purification

Full-length cDNAs of *OMTs* including *Cc6OMT1*, *Cc6OMT2*, *Ct7OMT*, *and CtSOMT* were obtained by PCR amplification using primers (Supplementary Table S5), and cloned into the pCzn1 vector (Zoonbio Biotechnology, China). The vector was introduced into the *Escherichia coli* line Arctic-Express (Zoonbio Biotechnology, China) for protein expression. The expression of the recombinant protein was induced by 0.5 mM of IPTG at 37^°^C for 4 h. The cells were harvested by centrifugation and resuspended in binding buffer, and the suspension was subsequently homogenized by 1 h of 200Wsonication (Vibra Cell VC 505 Sonicator; Sonics & Materials, Newtown, CT, United States). Cell debris was subsequently removed with 10-min centrifugation at 12,000 rpm. The purified inclusion body fist were denatured with 2 and 8 M urea, then renatured using 0.15 M NaCl. The protein was purified by Ni-IDA-Sepharose CL-6B (Spectrum Chemical Manufacturing, United States) under the manufacturer’s instructions. The purity of the His-tagged protein was determined by SDS–PAGE followed by Coomassie Brilliant Blue staining.

### Determination of Enzyme Activity

The standard enzyme assay for OMTs activity was performed using a reaction mixture in 50 μl of 25 mM Tris–HCl (pH 8.0), 25 mM sodium ascorbate, 0.1 mM *S*-adenosyl-L-methionine, 100 μM potential alkaloid substrate, and 50 μg of purified recombinant enzyme. Assays were carried out at 30^°^C for 1 h and terminated by adding 200 μL of ethanol. Controls were performed with denatured purified His-tagged proteins prepared by boiling in water for 20 min (Robin et al., 2016). Products were centrifuged at 14,000 rpm for 10 min, and 2 μL supernatants were subjected to UPLC-QTOF-MS/MS system (Waters Corporation, Milford, MA, United States), which consisted of a UPLC I-Class instrument (Waters Corporation, Singapore). UPLC was carried out using a Waters Acquity UPLC HSS T3 C18 column (2.1 mm × 100 mm, 1.8 μm) at a flow rate of 0.5 mL⋅min^-1^, and the temperature was set at 35^°^C. The mobile phases were 0.1% formic acid in H_2_O (A) and acetonitrile (B), with the following gradient: 2% B (0–1.5 min), 2–30% B (1.5–4 min), 30–60% B (4–6 min), 60–80% B (6–7.5 min), 80% B (7.5–8 min), and 2% B (8–10 min). Mass spectrometry was performed on a definition accurate mass quadrupole time-of-flight (Q-TOF) Xevo G2-S mass spectrometer (Waters MS Technologies, Manchester, United Kingdom) equipped with electrospray ionization (ESI) source. Eluate was applied to the mass analyzer using an electrospray ionization interface operating in positive mode with the following conditions: capillary voltage of 3.0 kV, cone voltage of 40 V, source temperature of 100^°^C, desolvation temperature of 400^°^C, cone gas flow of 50 L/h, and desolvation gas flow of 700 L/h. The energy for collision-induced dissociation (CID) was set to 6 V for the precursor ion, and the MS/MS fragment information was obtained using a collision energy ramp from 35 to 50 eV in MS^E^ mode. To ensure the mass accuracy and reproducibility of the optimized MS condition, leucine encephalin (m/z 556.2771 in positive mode) was used as the reference lock mass (Yang et al., 2015). Compounds were identified based on retention times and ESI [+]-CID spectra compared with authentic standards. Authentic (*S*)-norcoclaurine, (*S*)-coclaurine, (*S*)-scoulerine and (*S*)-tetrahydrocolumbamine were purchased from JK chemical (Beijing, China).

### Reverse Transcription Quantitative Real-Time PCR Analysis

Total RNA isolated from leaves, roots and rhizomes of the two *Coptis* species was used for reverse transcription quantitative real-time PCR (qRT-PCR) analysis. cDNA was transcribed from 2 μg purified RNA using HiScript QRT SuperMix (Vazyme, Nanjing, China). 2 μL (100 ng/μL) of cDNA in 20 μL solution system composed of 2 × SYBR Green Master mix (TaKaRa) was used for gene expression analysis in a Roche LightCycler 2.0 system (Roche Applied Science, Branford, CT, United States). The primers used in this study are listed in Supplementary Table S6. PCR amplification conditions were: 95^°^C for 30 s; 40 cycles of 95^°^C for 20 s, 55^°^C for 20 s and 72^°^C for 30 s. The actin gene was chosen as a reference gene to control for normalization. The relative changes in gene expression levels were calculated using the 2^-ΔΔCt^ method. For each target gene, the experiment was carried out with three biological replicates.

### Statistical Analysis

Data shown are the mean ± standard deviation of three biological replicates. Mean differences were compared using the statistical software data processing system (SPSS 17.0). The significant differences between samples were statistically evaluated by the Students’s *t*-test for HPLC and Tukey test for qRT-PCR.

## Results and Discussion

### Morphology and Quantitative Analysis of Major BIAs in Two *Coptis* Species

**FIGURE 2:**
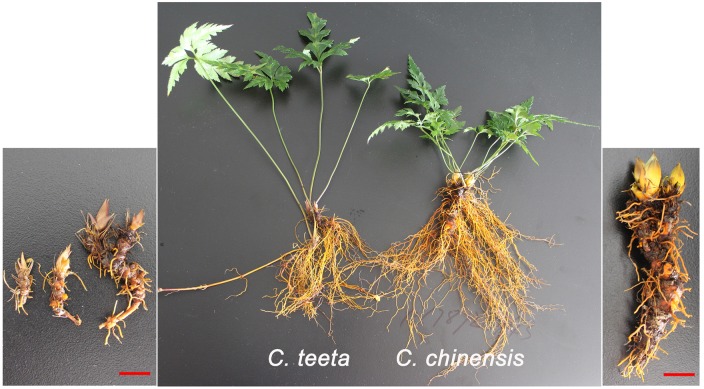
Morphology comparison of roots and shoots between *C. teeta* and *C*. *chinensis.* Three-year-old *C. teeta* and *C*. *chinensis* were collected from Yunnan and Guangxi, respectively.

While the distribution of *C. teeta* is restricted to high-altitude regions, including Yunnan Province in China, *C. chinensis* is widely distributed in China. To differentiate *C. teeta* from *C. chinensis*, we randomly collected twenty plants of *C. teeta* and *C. chinensis*, respectively and compared their morphology. The aboveground of *C. teeta* is upright and compact with long petiole and more shoots, while that of *C. chinensis* is loose with short petiole and less shoots (**Figure 2**). In contrast, the belowground of *C. teeta* has small rhizomes and less roots but contains foraging branches, while *C. chinensis* has large rhizomes and more roots but has no foraging branches (**Figure 2**). Foraging branches (vegetative propagules) are mainly used for asexual reproduction, which is an adaptation to high altitudes as the environment of these regions is unfavorable for sexual reproduction (Yang et al., 2013). Therefore, the morphological evolution of *C. teeta* coincides with its geographical distribution.

**FIGURE 3:**
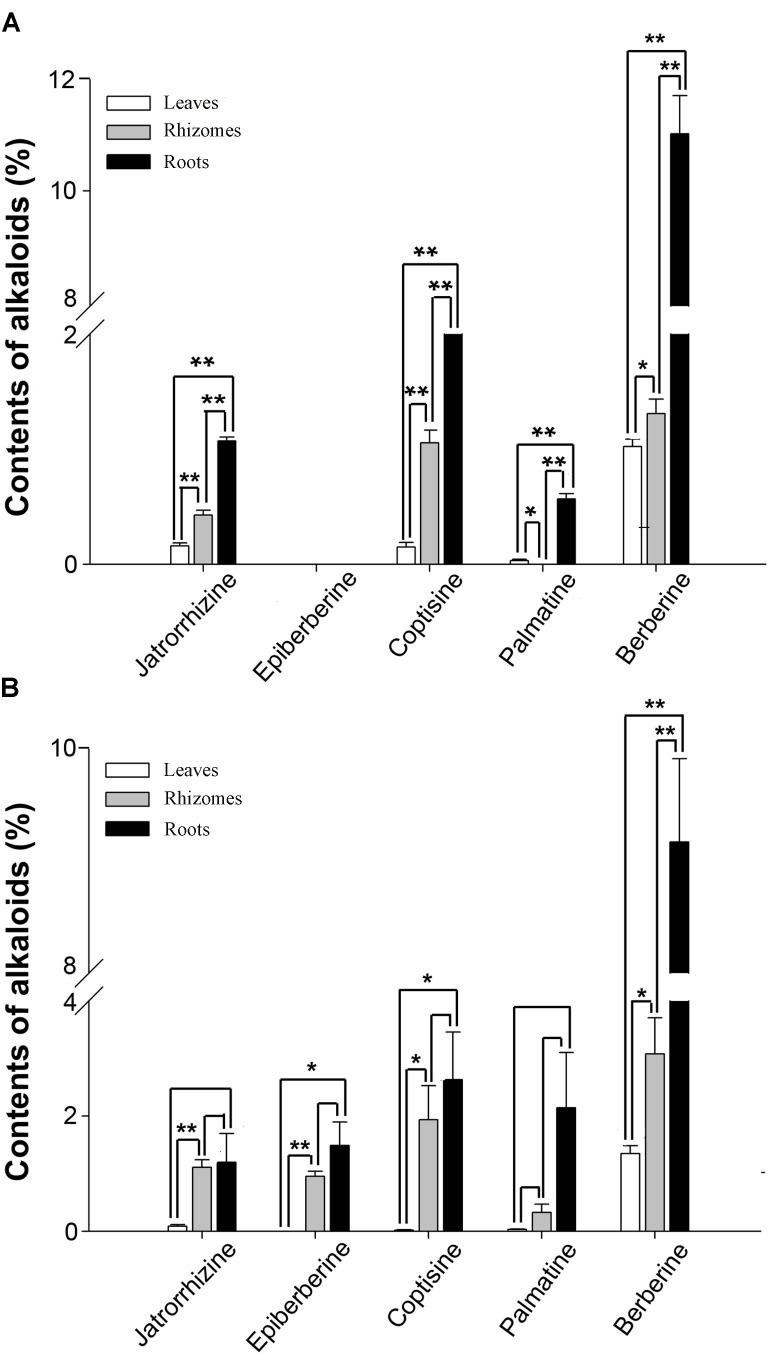
The contents of five main BIAs in three organs of *C. teeta* **(A)** and *C*. *chinensis* **(B)**. ^∗^*P* < 0.05, ^∗∗^*P* < 0.01.

According to previous reports, berberine, coptisine, jatrorrhizine, and palmatine constitute the main BIAs of *C. teeta* and *C. chinensis* (Kamath et al., 2009; Chen et al., 2017; Yang et al., 2017). HPLC analysis of leaves, roots, and rhizomes of *C. teeta* and *C. chinensis* indicated the presence of berberine, coptisine, jatrorrhizine, and palmatine in both species (**Figure 3**). The predominant BIA was berberine, followed by coptisine, and jatrorrhizine and palmatine was the lowest among them. Notably, epiberberine was only detected in *C. chinensis*. In terms of different tissues, the four major BIAs were most abundant in the roots, followed by rhizomes and leaves, which is consistent with the use of roots in TCM. In leaves, there were no significant differences between berberine and palmatine in two *Coptis* species, while coptisine and jatrorrhizine were 8 and 2 times higher in *C. teeta* than that in *C. chinensis*, respectively. In rhizomes, berberine, coptisine, and jatrorrhizine were 2 times higher in *C. chinensis* than that in *C. teeta*. Morever, palmatine was detected only in *C. chinensis*. In roots, there were no significant differences among berberine, coptisine and jatrorrhizine in two *Coptis* species, whereas palmatine was 4 times higher in *C. chinensis* than that in *C. teeta* (**Figure 3**). The results of the present study thus further illustrate the similarities and differences in BIA contents and types between *C. teeta* and *C. chinensis*. Such differences may explain the reported variations in pharmacological activities between the two species (Feng et al., 2011). Further studies on their pharmacological activity will also be helpful in providing a scientific basis for using a particular *Coptis* species for treating a specific condition.

### Illumina Sequencing, *de Novo* Assembly, and Functional Annotation

To obtain a comprehensive *C. teeta* and *C*. *chinensis* transcriptome, 12 cDNA libraries, CtLF| CtRT and CcLF| CcRT, were constructed from total RNA extracted from the roots and leaves of two *Coptis* species, respectively. Three biological replications for each tissue were sequenced using the Illumina HiSeq 2000 sequencing platform. After removal of the adaptor sequences, ambiguous and low-quality reads (Q20 < 20), a total of 282,639,316 clean reads (total length: 39,748,135,308 bp; 39.7 Gb) and 312,917,026 clean reads (total length: 43,953,381,053; 44.0 Gb) were respectively generated. The Q20 (sequencing error rate < 1%) and GC percentages of CtLF| CtRT were 98.3 and 43.82%, respectively, and those of CcLF| CcRT were 98.33 and 43.66%, respectively (Supplementary Table S1). With the aid of the short-reads assembly software Trinity (Grabherr et al., 2011), the clean reads of the CtLF| CtRT cDNA libraries were *de novo* assembled into 81,823 unigenes, with an N50 length of 1,536 bp and a mean length of 810 bp (Supplementary Table S1, Supplementary Figure S1, and Supplementary Data S1). The other CcLF| CcRT cDNA libraries generated a total of 78,499 unigenes, with an average length of 784 bp and an N50 length of 1,491 bp (Supplementary Table S1, Supplementary Figure S1, and Supplementary Data S2). The high-quality reads obtained in this study were deposited in the NCBI SRA database (accession number SRA588794).

To annotate the assembled unigenes, the sequences were searched against four public protein databases: NR, Swiss-Prot, KOG, and KEGG. A total of 38,398 unigenes (46.93%) in *C. teeta* and 36,745 unigenes (46.81%) in *C. chinensis* were annotated in the public databases. Additionally, in *C. teeta*, 34,888 (42.64%), 29,615 (36.19%), 17,444 (21.32%), and 26,109 (31.91%) unigenes were annotated in the NR, Swiss-Prot, KEGG, and KOG databases, respectively. However, in *C. chinensis*, 34,273 (43.66%), 28,040 (35.72%), 15,992 (20.37%), and 24,285 (30.94%) unigenes were annotated in the NR, Swiss-Prot, KEGG, and KOG databases, respectively (Supplementary Table S2).

In the GO analyses, 23,066 unigenes from *C. teeta* were classified into three classes, including cellular components (9,155 unigenes), biological processes (9,371 unigenes), and molecular functions (4,540 unigenes). In *C. chinensis*, 26,005 unigenes were classified into three categories; namely, cellular components (11,046 unigenes), biological processes (10,108 unigenes), and molecular functions (4,851 unigenes) (Supplementary Figure S2). Approximately 40,493 and 37,121 unigenes from *C. teeta* and *C. chinensis* were respectively annotated and grouped into 24 KOG categories. In both *C. teeta* and *C. chinensis*, the largest cluster was general function prediction, which is related to only basic physiological and metabolic functions and accounted for 8,921 unigenes (22.03%) and 8,373 unigenes (22.56%), respectively. However, the smallest category was cell motility, which represented 0.03% of the annotated unigenes (Supplementary Figure S3). KEGG pathway analysis is helpful for understanding the biological functions and interactions of genes. A total of 5,894 unigenes from *C. teeta* were annotated in the KEGG database and assigned to 124 biological pathways. The largest pathway was the metabolic pathway, which consisted of 4,917 unigenes, included biosynthesis of other secondary metabolites (333 unigenes), which included BIA biosynthesis. In contrast, a total of 7,285 unigenes were annotated in the KEGG database and were assigned to 124 biological pathways in *C. chinensis*. Similar to *C. teeta*, the metabolic pathway that contained 6,066 unigenes was the largest one, included the biosynthesis of other secondary metabolites (406 unigenes) (Supplementary Figure S4).

### Transcripts Encoding Enzymes Involved in BIA Biosynthesis

Benzylisoquinoline alkaloid synthesis starts from tyrosine, which forms the precursor (*S*)-reticuline via a multi-step enzymatic reaction. (*S*)-reticuline is then transformed into different proberberines, including berberine, coptisine, palmatine, columbamine, and magnoflorine. To date, biosynthetic pathways for berberine, palmatine, coptisine, columbamine, and magnoflorine have been characterized in other plant species (Ikezawa et al., 2008; Ziegler et al., 2009; Beaudoin and Facchini, 2014; Hagel et al., 2015). However, pathways for jatrorrhizine and epiberberine have not been determined to date. On the basis of previous reports and present transcriptome data, we hereby propose the biosynthetic pathways for berberine, palmatine, jatrorrhizine, coptisine, columbamine, magnoflorine, and epiberberine (Beecher and Kelleher, 1983; Rüffer et al., 1983) (**Figure 1**). According to the KEGG pathway, we discovered the transcripts encoding almost all the known enzymes for (*S*)-reticuline biosynthesis in our Illumina dataset, which included L-tyrosine aminotransferase (TyrAT), tyrosine decarboxylase (TYDC), tyrosine/tyramine 3-hydroxylase (3OHase), NCS, 6OMT, CNMT, NMCH, and 4^′^OMT (**Table 1** and Supplementary Tables S3, S4). Berberine is widely distributed in plants and has been documented intensively (Sato and Yamada, 1984; Ikuta and Itokawa, 1988; Hagel and Facchini, 2013; Sato, 2013). Thus, several genes encoding proteins associated with berberine biosynthesis have been identified and characterized. These include the berberine bridge enzyme (BBE; Bird et al., 2003; Minami et al., 2008), which converts (*S*)-reticuline to berberine, SOMT (Takeshita et al., 1995), (*S*)-canadine synthase (CAS; Ikezawa et al., 2003), and (*S*)-tetrahydroprotoberberine oxidase (STOX; Matsushima et al., 2012; Sato, 2013). However, the pathway of epiberberine, which is closely related to berberine in structure, has not been determined. We propose that scoulerine is subsequently *O*-methylated at C2, which forms methylenedioxy bridges and undergoes oxidation to yield epiberberine, which is catalyzed by members of the OMT, CYP719, and OX families that consist of 18, 4, and 2 candidate genes, respectively. The coptisine, palmatine, columbamine, and magnoflorine biosynthesis pathways have previously been described as follows. For all of these pathways, (*S*)-scoulerine is first formed from (*S*)-reticuline by BBE (Facchini et al., 1996; Winkler et al., 2006). The conversion of (*S*)-scoulerine to coptisine begins with the formation of two methylenedioxy bridges by cheilanthifoline synthase (CFS) and stylopine synthase (SPS), which are members of the CYP719 family, forming (*S*)-stylopine. CFS and SPS have been isolated from *E. californica* (Ikezawa et al., 2009; Mizutani and Sato, 2011) and *Argemone mexicana* (Chavez et al., 2011) and characterized. Subsequent oxidation of (*S*)-stylopine by STOX yields coptisine (Matsushima et al., 2012). (*S*)-scoulerine is sequentially converted to (*S*)-tetrahydrocolumbamine by SOMT, to columbamine by STOX, and to palmatine by columbamine *O*-methyltransferase (CoOMT) (Pienkny et al., 2009; Ziegler et al., 2009). In magnoflorine biosynthesis, (*S*)-reticuline is first oxidized to (*S*)-corytuberine by (*S*)-corytuberine synthase (CTS), and subsequently, (*S*)-corytuberine-*N*-methyltransferase (SCNMT) converts (*S*)-corytuberine to magnoflorine (Ikezawa et al., 2008) (**Table 1**, **Figure 1**, and Supplementary Tables S3, S4).

**Table 1:** Unigenes involved in benzylisoquinoline alkaloid biosynthesis in *C. teeta* and *C. chinensis*.

Gene name (EC number)	*C. teeta*	*C. chinensis*
TYDC, tyrosine decarboxylase (4.1.1.25)	5	4
3OHase, tyrosine/tyramine 3-hydroxylase (1.14.16.2)	6	7
TyrAT, L-tyrosine aminotransferase (2.6.1.5)	2	3
4HPPDC, 4-hydroxyphenylpuruvate decarboxylase (4.1.1.80)	0	0
NCS, (*S*)-norcoclaurine synthase (4.2.1.78)	14	14
6OMT, (*S*)-norcoclaurine 6-*O*-methyltransferase (2.1.1.128)	2	3
CNMT, (*S*)-coclaurine *N*-methyltransferase (2.1.1.140)	5	4
NMCH, *N*-methylcoclaurine 3^′^-monooxygenase (1.14.13.71)	2	1
4^′^OMT, 3^′^-hydroxy-*N*-methyl-(*S*)-coclaurine 4^′^-*O*-methyltransferase (2.1.1.116)	2	1
BBE, berberine bridge enzyme (1.21.3.3)	8	8
SOMT, (*S*)-scoulerine 9-*O*-methyltransferase (2.1.1.117)	1	1
CAS, (*S*)-canadine synthase (1.14.21.5)	2	2
STOX, (*S*)-tetrahydroprotoberberine oxidase (1.3.3.8)	1	1
CTS, (*S*)-corytuberine synthase (1.14.21.-)	1	1
CoOMT, columbamine *O*-methyltransferase (2.1.1.118)	2	2
CFS, (*S*)-cheilanthifoline synthase (1.14.21.2)	0	0
SPS, (*S*)-stylopine synthase (1.14.21.1)	0	0
**All number of unigenes**	**53**	**52**

**FIGURE 4:**
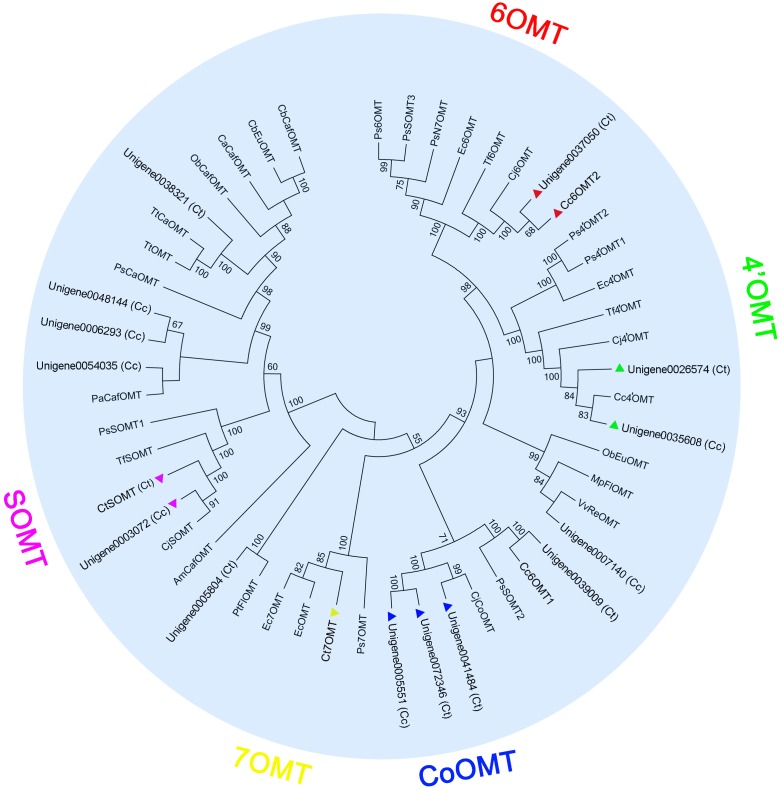
Phylogenetic tree of OMTs. Phylogenetic tree was constructed based on the deduced amino acid sequences from *C. teeta* and *C*. *chinensis* OMTs (triangles of different colors) and other plant OMTs. Abbreviations and GenBank accession numbers for the sequences used are as follows: EcOMT, putative *E. californica* OMT (ACO90220.1); CjCoOMT, *C. japonica* columbamine OMT (Q8H9A8.1); TtOMT, *Thalictrum tuberosum* catechol OMT (AAD29845.1); TtCaOMT, *T. tuberosum* catechol OMT (AAD29843.1); PsCaOMT, *P. somniferum* catechol OMT (AAQ01670.1); PsN7OMT, *P. somniferum* norreticuline 7OMT (ACN88562.1); Ps6OMT, *P. somniferum* norcoclaurine 6OMT (AAP45315.1); Ps4^′^OMT2, *P. somniferum* 3^′^-hydroxy-*N*-methylcoclaurine 4^′^OMT2 (AAP45314.1); Ps4^′^OMT1, *P. somniferum* 3^′^-hydroxy-*N*-methylcoclaurine 4^′^OMT1 (AAP45314.1); Cc4^′^OMT, *C. chinensis* 3^′^-hydroxy-*N*-methylcoclaurine 4^′^OMT (ABY75613.1); Cj4^′^OMT, *C. japonica* 3^′^-hydroxy-*N*-methylcoclaurine 4^′^OMT (Q9LEL5.1); Tf4^′^OMT, *T. flavum* 3^′^-hydroxy-*N*-methylcoclaurine 4^′^OMT (AAU20768.1); Cj6OMT, *C. japonica* norcoclaurine 6OMT (Q9LEL6.1); Tf6OMT, *T. flavum* norcoclaurine 6OMT (AAU20765.1); VvReOMT, *Vitis vinifera* resveratrol OMT (CAQ76879.1); PtFlOMT, *Populus trichocarpa* flavonoid OMT predicted protein (XP_002312933.1); CjSOMT, *C. japonica* scoulerine 9OMT (Q39522.1); TfSOMT, *T. flavum* scoulerine 9OMT(AAU20770.1); PaCafOMT, *Picea abies* caffeate OMT (CAI30878.1); CaCafOMT, *Capsicum annuum* caffeate OMT (AAG43822.1); PsOMT1, *P. somniferum* SOMT1 (JN185323); PsOMT2, *P. somniferum* SOMT2 (JN185324); PsOMT3, *P. somniferum* SOMT3 (JN185325); ObEuOMT, *Ocimum basilicum* eugenol OMT (AAL30424.1); MpFlOMT, *Mentha X piperita* flavonoid 8OMT (AAR09600.1); ObCafOMT, *O. basilicum* caffeate OMT (AAD38189.1); CbEuOMT, *Clarkia breweri* (iso)eugenol OMT (AAC01533.1); CbCafOMT, *C. breweri* caffeate OMT (O23760.1); AmCafOMT, *Ammi majus* caffeate OMT (AAR24095.1); Ec7OMT, *E. californica* reticuline 7OMT (BAE79723.1); Ps7OMT, *P. somniferum* reticuline 7OMT (AAQ01668.1); Ec4^′^OMT, *E. californica* 3^′^-hydroxy-N-methylcoclaurine 4^′^OMT (BAM37633.1); and Ec6OMT, *E. californica* OMT (BAM37634.1).

Structurally, jatrorrhizine is a special protoberberine because it contains an unusual 7-*O*-methylation pattern. Previous studies have suggested that jatrorrhizine is synthesized via ring-opening of berberine (Beecher and Kelleher, 1983; Rüffer et al., 1983) (**Figure 1**, pathway 1). However, the enzyme catalyzing this ring-opening reaction has not been investigated to date. Six-*O*-methyltransferase catalyzes the formation of 6-*O*-methylnorlaudanosoline accompanied by a smaller amount of 7-*O*-methylnorlaudanosoline, which initiates its biosynthesis using norlaudanosoline instead of reticuline; however, testing for the relevant enzyme activity has not been conducted (Beecher and Kelleher, 1983; Rüffer et al., 1983). In the present study, we obtained 18 unigenes encoding OMT from our transcriptome data. Phylogenetic tree analysis revealed that unigenes 0037050 (Ct) and 0033238 (Cc) (designated as *Cc6OMT2*, MH165876) had high sequence identity with Cj6OMT, whereas unigenes 0026574 (Ct) and 0035608 (Cc) were clustered closely with 4^′^OMTs from *C. coptis* and other plants. Unigenes 0042287 (Ct) (designated as *CtSOMT*, MH165874) and 0003072 (Cc) had high sequence homology with CjSOMT, whereas unigenes 0041484 (Ct), 0072346 (Ct), and 0005551 (Cc) had high sequence homology with CjCoOMT. Unigene 0046714 (Ct) (designated as *Ct7OMT*, MH165877) clustered closely with 7OMT, and eight other OMTs [unigenes 0042981 (Cc) (designated as *Cc6OMT1*, MH165875), 0039009 (Ct), 0007140 (Cc), 0005804 (Ct), 0054035 (Cc), 0006293 (Cc), 0048144 (Cc), and 0038321 (Ct)] were clustered together with different known OMTs (**Figure 4**).

### Functional Characterization of OMT Candidates

**FIGURE 5:**
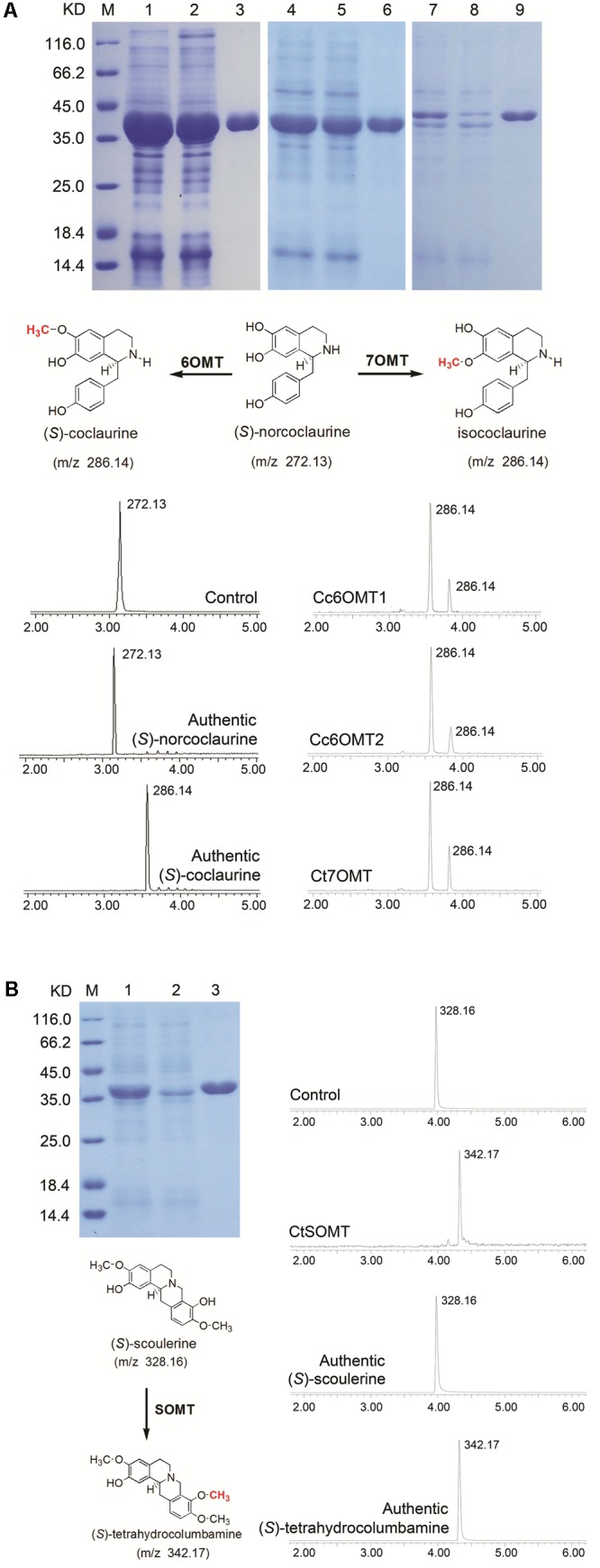
Extract ion chromatograms showing the *O*-methylation activity of recombinant OMTs on various substrates. **(A)** His-tag purified recombinant OMTs on 12% SDS–PAGE gel, Lane M: Protein Marker, Lane 1: Unpurified *Cc6OMT1, Lane 2: Flow through Cc6OMT1, Lane 3: Elution Cc6OMT1; Lane 4: Unpurified Cc6OMT2, Lane 5: Flow through Cc6OMT2, Lane 6: Elution Cc6OMT2; Lane 7: Unpurified Ct7OMT, Lane 8: Flow through Ct7OMT, Lane 9: Elution Ct7OMT. Verified reaction equation, the top (control) substrate with denatured purified OMTs proteins, *in vitro* assay products of Cc6OMT1, Cc6OMT2, Ct7OMT with (*S*)-norcoclaurine, respectively. **(B)** His-tag purified recombinant OMT on 12% SDS–PAGE gel, Lane M: Protein Marker, Lane 1: Unpurified CtSOMT, Lane 2: Flow through CtSOMT, Lane 3: Elution CtSOMT; verified reaction equation; the top (control) substrate with denatured purified OMT protein, *in vitro* assay product of CtSOMT with (*S*)-scoulerine. Products were identified based on retention times and ESI[+]-CID spectra using authentic standards, 1. (*S*)-norcoclaurine, 2. (*S*)-coclaurine, 3. (*S*)-scoulerine, 4. (*S*)-tetrahydrocolumbamine.*

Jatrorrhizine is structurally similar to berberine, functions in detoxification and imparts bactericidal properties and hypoglycemic effects (Arens et al., 1985; Moody et al., 1995; Volleková et al., 2003); however, its biosynthetic pathway remains unclear. Molecular structural analysis indicates that jatrorrhizine contains an unusual 7-*O*-methylation pattern that makes it difficult to deduce its biosynthesis from reticuline, the common precursor of BIAs. Previous studies involving ^14^C isotopic tracers have shown that 6OMT catalyzes the formation of 6-*O*-methylnorlaudanosoline along with a smaller amount of 7-*O*-methylnorlaudanosoline; thus, it is probable that the 7-*O*-methylation pattern of jatrorrhizine is already established at the norlaudanosoline step (Beecher and Kelleher, 1983; Rüffer et al., 1983). In the present study, five 6OMT unigenes; namely, 0039009 (Ct), 0037050 (Ct), 0071836 (Cc), Cc6OMT1, Cc6OMT2, and one 7OMT unigene, Ct7OMT, were selected to study their function by *in vitro* enzyme activity analysis. **Figure 5A** shows that incubation of Cc6OMT1, Cc6OMT2 and Ct7OMT with (*S*)-norcoclaurine (mass-to-charge ratio [m/z] 272.13) yielded two peaks with m/z 286.14 at 3.55 min and 3.83 min, respectively. A molecular formula (C17H20NO3) was obtained by their accurate molecular weight and element composition analysis. One product with m/z 286.14 at 3.55 min was identified as (*S*)-coclaurine by comparison of retention time and mass spectra with authentic standard. Based on accurate molecular weight and molecular formula, another product with m/z 286.14 at 3.83 min was proposed as isococlaurine which contains a 7-*O*-methylation pattern. This means that Cc6OMT1 and Cc6OMT2 are capable of *O*-methylating (*S*)-norcoclaurine at C6, yielding (*S*)-coclaurine along with a smaller amount of *O*-methylation at C7, thereby forming its isomer, isococlaurine, and Ct7OMT also *O*-methylates (*S*)-norcoclaurine at C6, yielding (*S*)-coclaurine, along with *O*-methylation of (*S*)-norcoclaurine at C7, yielding isococlaurine (**Figure 5A** and Supplementary Figure S5). Subsequently, isococlaurine can be further transformed into jatrorrhizine through a series of enzymatic reactions (**Figure 1**, pathway 2). This result validates the previous results of feeding experiments with distant single or doubly labeled precursors (Rüffer et al., 1983), and shows that jatrorrhizine can be formed by (*S*)-norcoclaurine C7 *O*-methylation, thereby providing a molecular basis for jatrorrhizine synthesis via (*S*)-norcoclaurine. Despite their names suggesting strict functions, characterized OMTs of BIAs often display diversify functions in different species. For example, *P. somniferum* 6OMT specifically 6-*O*-methylated (*R,S*)-norcoclaurine, while 6OMT also 7-*O*-methylated (*R,S*)-isoorientaline. 6OMT from *C. japonica* efficiently 6-*O*-methylated (*R,S*)-norcoclaurine, (*R,S*)-norlaudanosoline, and (*R,S*)-laudanosoline, but weakly *O*-methylated (*R,S*)-6-*O*-methylnorlaudanosoline, (*S*)-scoulerine, and (*R,S*)-coclaurine, clearly not at the already *O*-methylated 6-position in each case (Sato et al., 1993; Morishige et al., 2000; Chang et al., 2015). Therefore, it is acceptable that Cc6OMT from *C. chinensis* was able to 6-*O*-methylate (*S*)-norcoclaurine, yielding (*S*)-coclaurine along with a smaller amount of *O*-methylation at C7. For 7OMT, *P. somniferum* 7OMT 7-*O*-methylated (*R,S*)-reticuline, (*R,S*)-orientaline, and (*R,S*)-protosinomenine, (*R,S*)-isoorientaline. Conversely, our present results showed that Ct7OMT from *C. teeta* mainly *O*-methylates (*S*)-norcoclaurine at C6, yielding (*S*)-coclaurine, along with *O*-methylation of (*S*)-norcoclaurine at C7, yielding isococlaurine that has been confirmed for the first time in all species. Therefore, we suggest that Ct7OMT should be more properly referred to as 6-/7- *O*-methyltransferase (Ct6/7OMT), which may be related to the particularity of *C. teeta*. Overall, depending on different species, both 6OMT and 7OMT often display a range of substrate, which contribute overlapped and divergent functions.

Furthermore, previous studies have shown that SOMT1 sequentially 9- and 2-*O*-methylates (*S*)-scoulerine, yielding (*S*)-tetrahydrocolumbamine and (*S*)-tetrahydropalmatine, respectively (Dang and Facchini, 2012). By comparing their molecular structures, we propose that epiberberine is synthesized through *O*-methylation of (*S*)-scoulerine at C2 (**Figure 1**). Thus, two unigenes, CtSOMT and 0003072 (Cc), were selected to study their enzyme activity. The results showed incubation of CtSOMT with (*S*)-scoulerine (m/z 328.16) yielded only one reaction product identified as tetrahydrocolumbamine (m/z 342.17) based on authentic standards. It means that CtSOMT has stringent substrate specificity because 9OMT targets (*S*)-scoulerine, but it could not *O*-methylate (*S*)-scoulerine at C2 (**Figure 5B** and Supplementary Figure S6). This may be attributable to the fact that *C. chinensis* has no (*S*)-tetrahydropalmatine, thereby leading to the inability of SOMT to 2-*O*-methylate (*S*)-tetrahydrocolumbamine.

### Expression Patterns of Putative Genes Involved in BIA Biosynthesis

**FIGURE 6:**
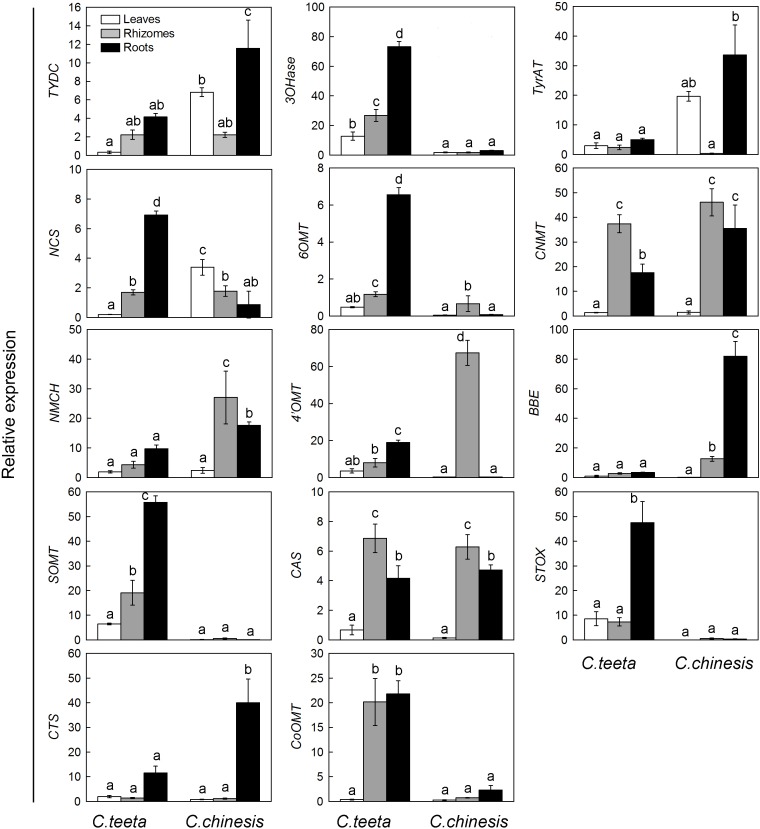
Expression patterns of candidate unigenes involved in BIA biosynthesis in *C. teeta* and *C*. *chinensis*. The transcripts were analyzed by qRT-PCR, with actin as an internal standard.

To investigate the expression patterns of candidate genes involved in BIA biosynthesis in two *Coptis* species, the expression levels of 14 unigenes related to protoberberine biosynthesis in the roots, rhizomes, and leaves of *C. teeta* and *C. chinensis* were performed by qRT-PCR. **Figure 6** indicates that most genes were upregulated in the roots rather than in the leaves of the two *Coptis* species. In *C. teeta*, the highest expression was observed in the roots and the lowest in the leaves, whereas some genes were more highly expressed in the rhizomes than in the roots in *C. chinensis*, such as *NCS, 6OMT, 4*^′^*OMT*, and *SOMT*. The enzymes encoded by the genes *NCS, 6OMT*, and *4*^′^*OMT* are located upstream of the BIA synthetic pathway from a key branch-point intermediate (*S*)-reticuline, whereas SOMT is involved in the synthesis of berberine and palmatine. The differential gene expression patterns of two *Coptis* species may have resulted from their growth environment, climate, topography, other environmental factors, and cultivation conditions (Chen et al., 2017). Moreover, these results coincided with the higher contents of major BIAs in the roots of the two *Coptis* species (**Figure 3**). BIA synthesis and accumulation in *Thalictrum* (Ranunculaceae) were in the pith and cortex of the roots and rhizomes (Samanani et al., 2005; Lee et al., 2013), suggesting that the main sites of BIA synthesis and accumulation in *C. chinensis* were probably in the roots.

## Conclusion

We conducted transcriptomic analysis of *C. teeta* and *C*. *chinensis* and obtained a total of 81,823 and 78,499 unigenes, respectively, which provide valuable genetic resources for these invaluable Chinese herb medicines. We propose the integrated biosynthetic pathways of berberine, palmatine, jatrorrhizine, coptisine, columbamine, magnoflorine, and epiberberine, and identified 53 and 52 unigenes involved in the biosynthesis of the protoberberine alkaloids in the two *Coptis* species *C. teeta* and *C*. *chinensis*, respectively. Further enzyme activity testing *in vitro* demonstrated that two 6OMTs and one 7OMT were able to 6-*O*-methylate (*S*)-norcoclaurine, yielding (*S*)-coclaurine along with a smaller amount of *O*-methylation at C7, whereas SOMT specifically catalyzed *O*-methylation at C2 on (*S*)-scoulerine, yielding (*S*)-tetrahydrocolumbamine. These results provide opportunities for the *de novo* production of active ingredients by microorganism engineering.

## Author’s Note

This paper is dedicated to Mrs. Li, Guanghui Zhang’s wife, who has been suffering from ataxia for many years. We are grateful to those who are willing to help her. Please contact zgh73107310@163.com.

## Author Contributions

WF and S-CY conceived the study. S-MH, Y-LL, KC, XW, YD, GC, J-JZ, Q-MZ, Z-LQ, J-LY, and XZ performed the experiments and carried out the analysis. S-MH, WF, S-CY, and G-HZ designed the experiments and wrote the manuscript.

## Conflict of Interest Statement

The authors declare that the research was conducted in the absence of any commercial or financial relationships that could be construed as a potential conflict of interest.

## Footnotes

1 http://www.ncbi.nlm.nih.gov/

2 http://www.expasy.ch/sprot/

3 http://www.ncbi.nlm.nih.gov/COG

4 http://www.genome.jp/kegg/
