# Supplementary material for: Identification and Characterization of Genes Involved in Benzylisoquinoline Alkaloid Biosynthesis in Coptis Species
Source: Front Plant Sci. 2018 Jun 4;9:731. doi: 10.3389/fpls.2018.00731 (PMC5995273; doi:10.3389/fpls.2018.00731)
Supplement: Supplementary file 1 [file Data_Sheet_1.DOC]

**Supplementary information for following article**

**Identification and characterization of genes involved in benzylisoquinoline alkaloid biosynthesis in two *Coptis* species**

Si-Mei He^1+^, Yan-Li Liang^1+^, Kun Cong^1^, Geng Chen^1^, Xiu Zhao^1^, Qi-Ming Zhao^1^, Jia-Jin Zhang^1^, Xiao Wang^2,4^, Yang Dong^3^, Jian-Li Yang^5^, Guang-Hui Zhang^1^, Zhi-Long Qian^1^, Wei Fan^1*^, Sheng-Chao Yang^1*^

^1^ State Key Laboratory of Conservation and Utilization of Bio-resources in Yunnan, National & Local Joint Engineering Research Center on Gemplasm Innovation & Utilization of Chinese Medicinal Materials in Southwest China, Yunnan Agricultural University, Kunming, China

^2^ State Key Laboratory of Genetic Resources and Evolution, Kunming Institute of Zoology, Chinese Academy of Sciences, Kunming, China

^3^ Province Key Laboratory, Biological Big Data College, Yunnan Agricultural University, Kunming, China

^4^ University of Chinese Academy of sciences, Beijing, China

^5^ State Key Laboratory of Plant Physiology and Biochemistry, College of Life Sciences, Zhejiang University, Hangzhou, China

^†^These authors have contributed equally to this work

***Corresponding authors:** Wei Fan, Sheng-Chao Yang

**Telephone number:** +86-871-65228927

**Fax number:** +86-871-65227712

**E-mail:** fanwei1128@aliyun.com, shengchaoyang@163.com


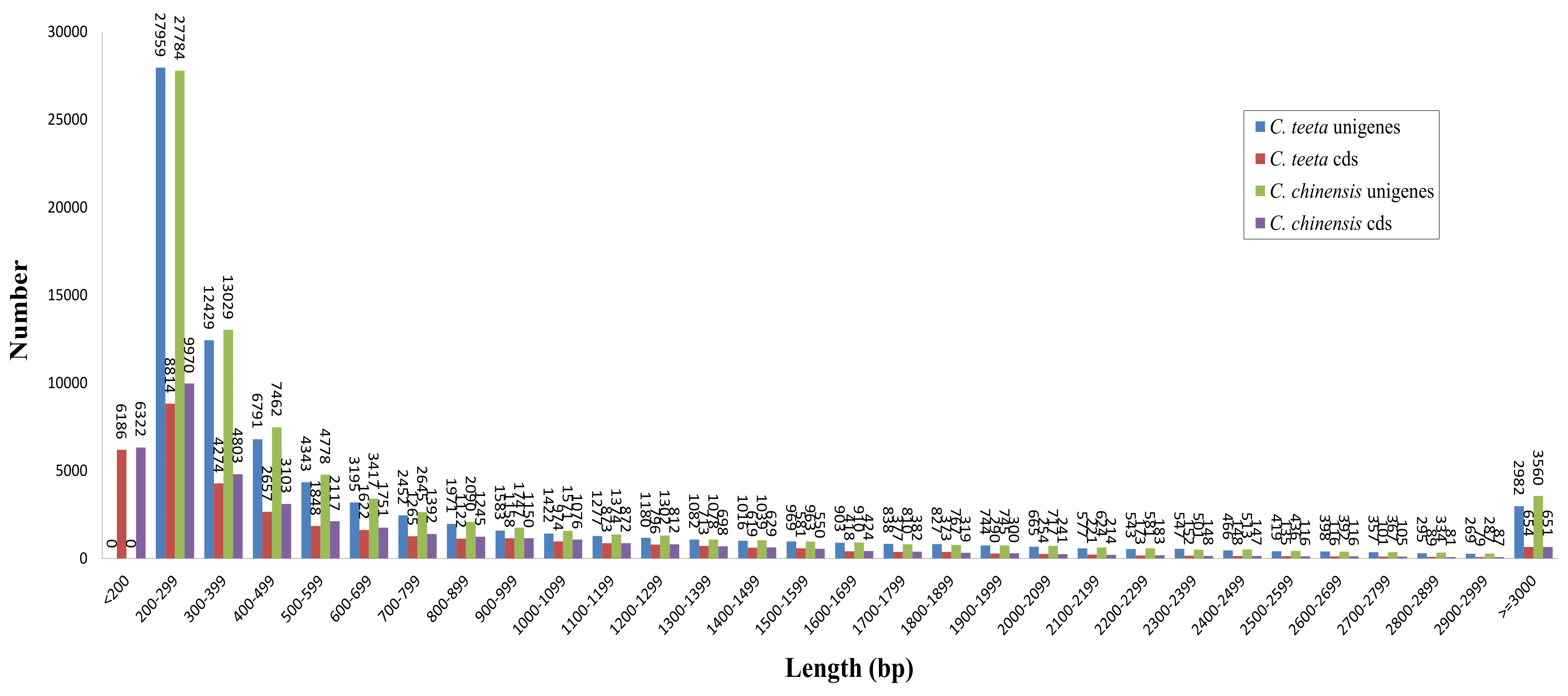


**Figure 1 Length distribution of unigenes and CDSs.**

**
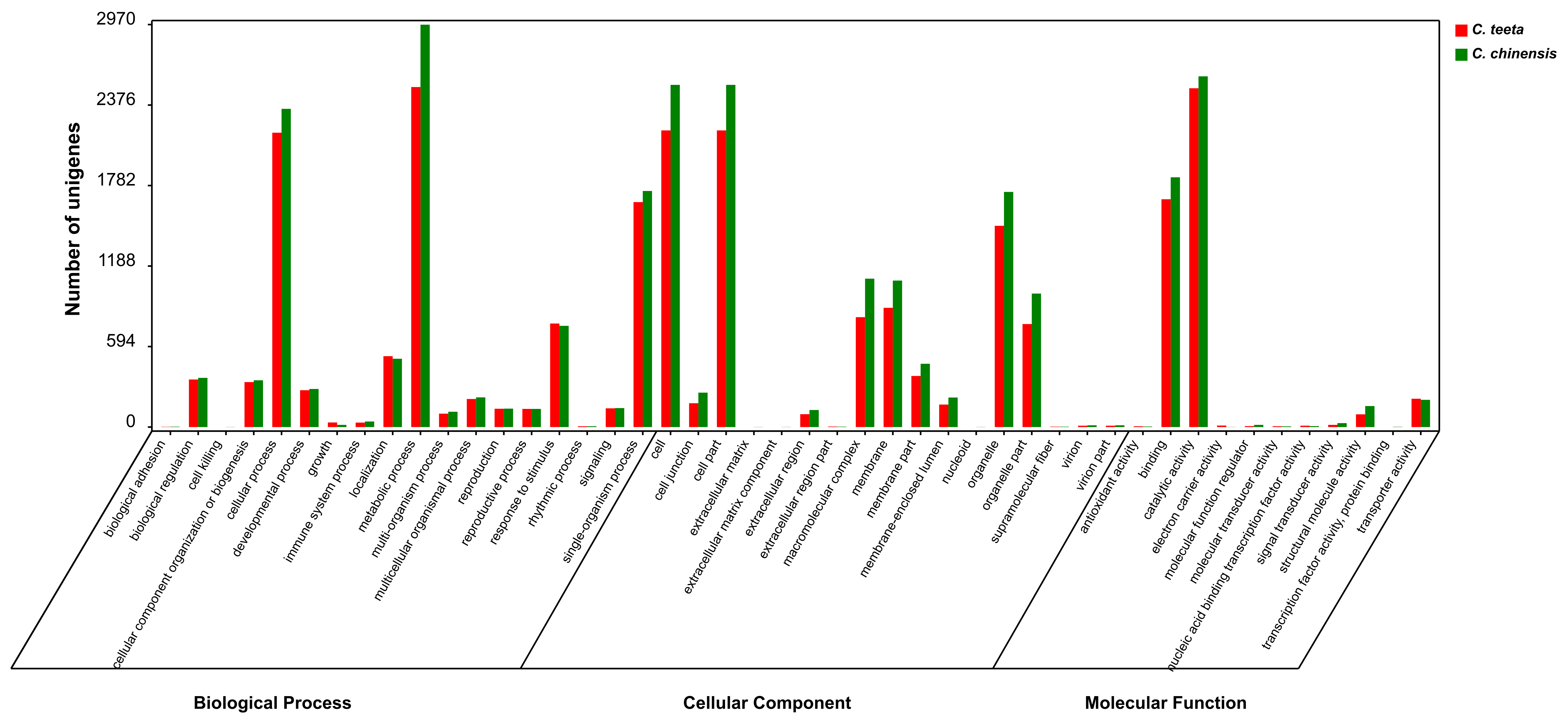
**

**Figure 2 GO classification of assembled unigenes.**

**
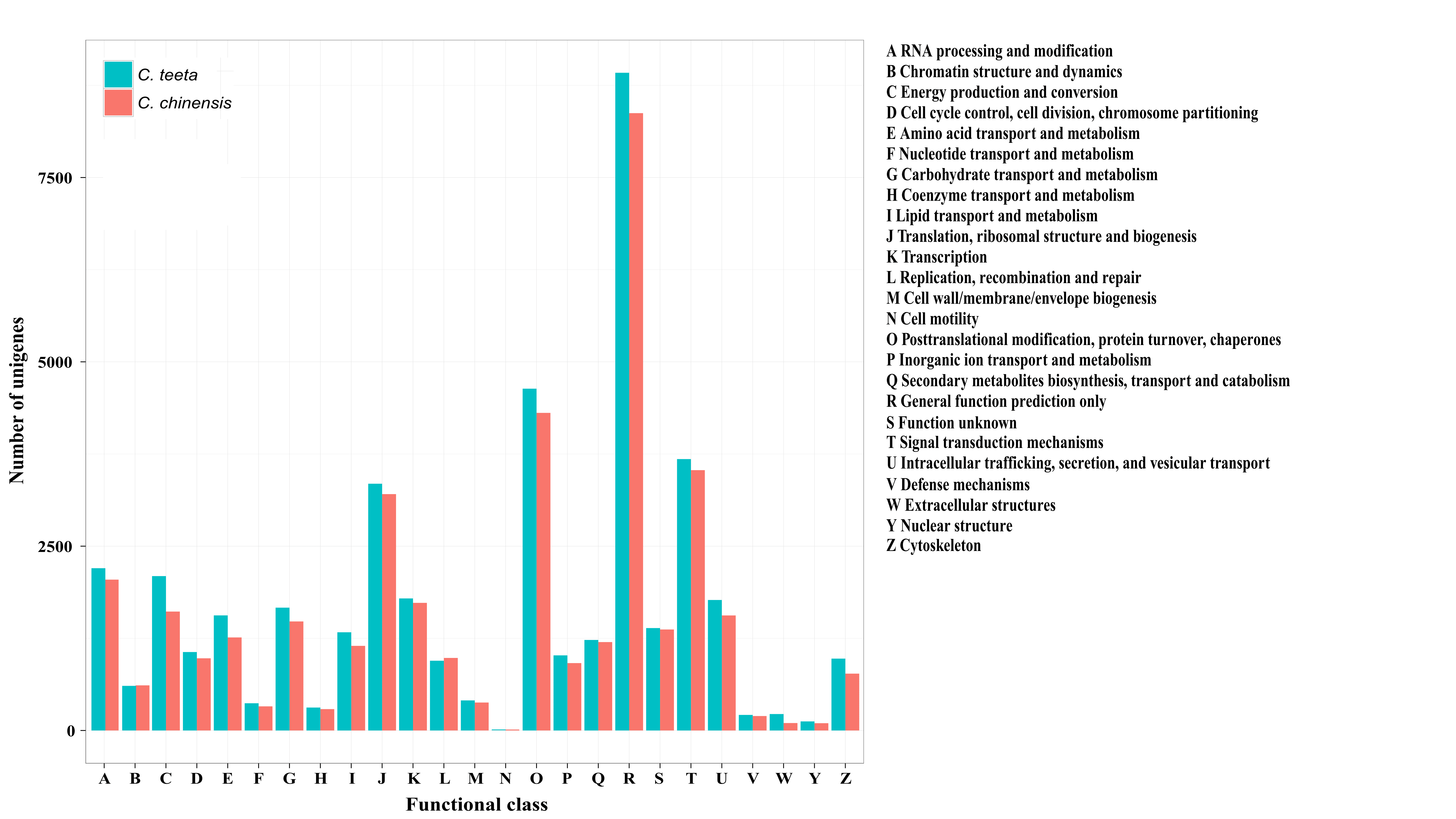
**

**Figure 3 KOG function classification of *C. teeta* and *C. chinensis* unigenes.**

**
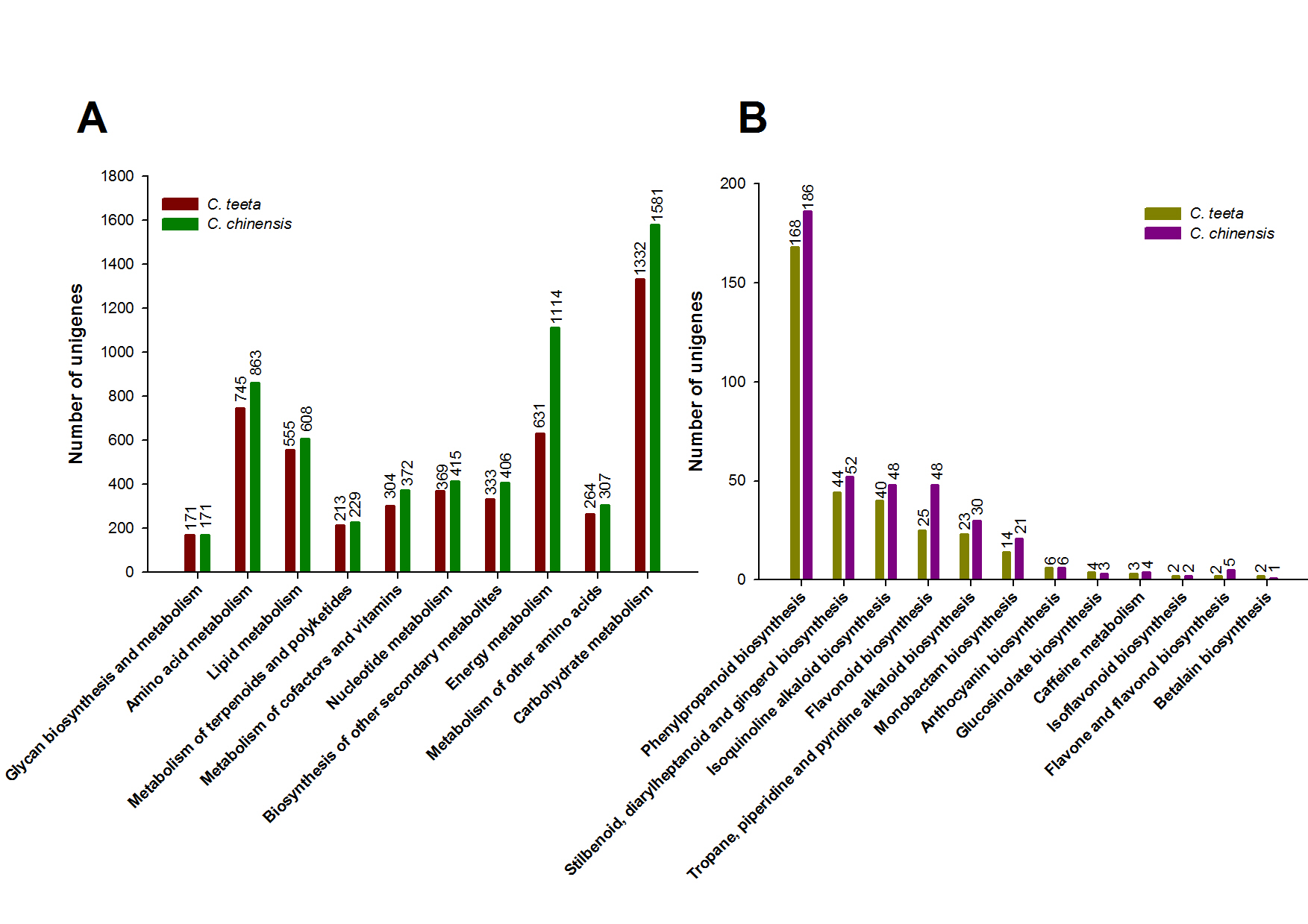
**

**Figure 4 Pathway assignment based on KEGG.** (A) Classification based on metabolism categories; (B) classification based on biosynthesis of other secondary metabolites.


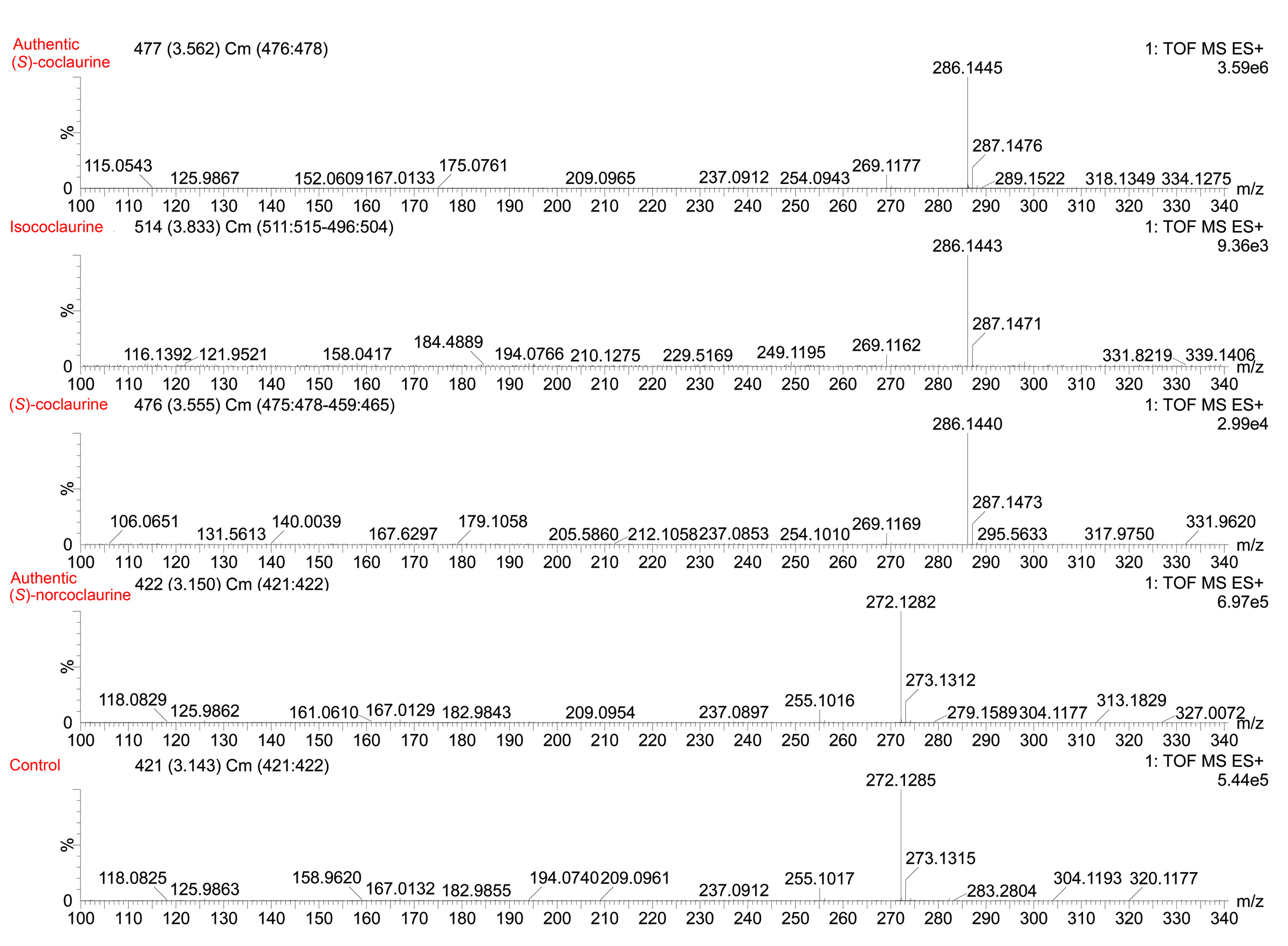


**Figure 5 QTOF-MS analysis of assay products of Cc6OMT1, Cc6OMT2, Ct7OMT with (*S*)-norcoclaurine in vitro, respectively.**

**
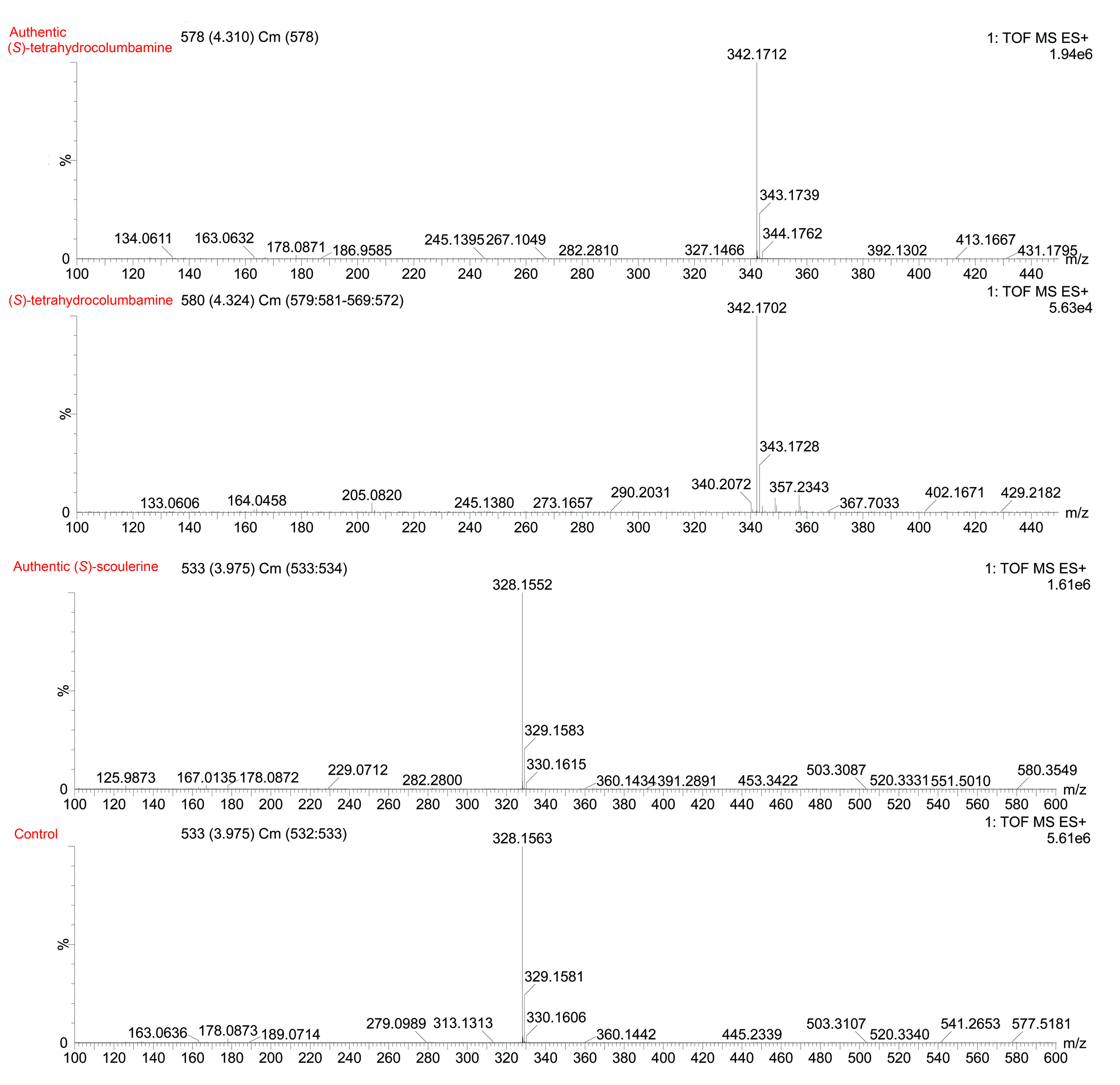
**

**Figure 6 QTOF-MS analysis of assay product of CtSOMT with (*S*)-scoulerine in vitro.**
